# A Survey on Sensor Coverage and Visual Data Capturing/Processing/Transmission in Wireless Visual Sensor Networks

**DOI:** 10.3390/s140203506

**Published:** 2014-02-20

**Authors:** Florence G. H. Yap, Hong-Hsu Yen

**Affiliations:** 1 English Division, Center for General Education, Chang Gung University, Taoyuan, 333, Taiwan; E-Mail: ghyap@mail.cgu.edu.tw; 2 Department of Information Management, Shih-Hsin University, Taipei, 116, Taiwan

**Keywords:** sensor coverage, visual data capturing, visual data processing, visual data transmission, wireless visual sensor networks

## Abstract

Wireless Visual Sensor Networks (WVSNs) where camera-equipped sensor nodes can capture, process and transmit image/video information have become an important new research area. As compared to the traditional wireless sensor networks (WSNs) that can only transmit scalar information (e.g., temperature), the visual data in WVSNs enable much wider applications, such as visual security surveillance and visual wildlife monitoring. However, as compared to the scalar data in WSNs, visual data is much bigger and more complicated so intelligent schemes are required to capture/process/transmit visual data in limited resources (hardware capability and bandwidth) WVSNs. WVSNs introduce new multi-disciplinary research opportunities of topics that include visual sensor hardware, image and multimedia capture and processing, wireless communication and networking. In this paper, we survey existing research efforts on the visual sensor hardware, visual sensor coverage/deployment, and visual data capture/processing/transmission issues in WVSNs. We conclude that WVSN research is still in an early age and there are still many open issues that have not been fully addressed. More new novel multi-disciplinary, cross-layered, distributed and collaborative solutions should be devised to tackle these challenging issues in WVSNs.

## 1. Introduction

**Figure 1.**
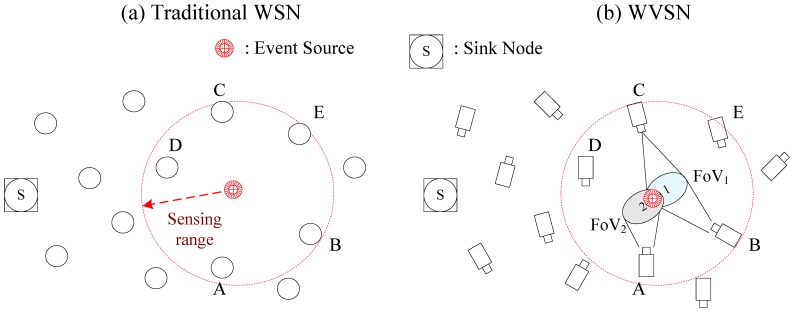
Wireless sensor networks *vs.* Wireless Visual Sensor Networks.

The wireless sensor networks (WSNs) represent a blooming technology where they can probe and collect environmental information, such as temperature, atmospheric pressure and irradiation to provide ubiquitous sensing, computing and communication capabilities. Besides collecting these scalar data (e.g., temperature) from the environment, a newer trend in WSNs is to deploy sensor nodes with cameras to capture and transmit visual data (i.e., images and video data) back to the sink node. Thanks to the rapid advancement of sensor technology, equipping sensors with cameras is possible [1]. In this way, sensor nodes can send the captured visual data to provide richer sensing and monitoring information, which enables more applications in areas such as wide-life observation and security surveillance. These kinds of camera-equipped sensor networks are known as Wireless Visual Sensor Networks (WVSNs). The hardware components of a WVSN consist of tiny camera sensor nodes, embedded processors and wireless transceivers [1]. The WVSN is totally different from a traditional WSN in five ways:

(1) *Field of View coverage requirement for data source nodes*: In traditional WSNs, when an event occurs, the nodes within the sensing range of the event will sense the event and become data source nodes to transmit the sensed data back to the sink. However, in WVSNs, besides the sensing range, another more important criterion, Field of View (FoV), should be considered. The FoV comes from the fact that the camera captures visual data of the event from a certain direction or angle. In other words, besides the sensing range coverage, the FoV of the sensor nodes should also cover the event so as to capture the visual data of event. In Figure 1, we illustrate an example to illustrate the idea of FoV. In Figure 1a, the event is inside the sensing range of the sensor nodes (i.e., nodes *A*, *B*, *C*, *D*, *E*) so they can sense the event and then transmit the sensed data back to the sink in the WSN. In Figure 1b, we can see that due to the angle of the camera on the sensors, not every sensor node inside the sensing range can capture the event. In Figure 1b, nodes *A*, *B* and *C* are the only data source nodes that can capture the visual data. In other words, even though nodes *D* and *E* are within the sensing range, the FoV of the cameras do not cover the event so that nodes *D* and *E* are not data source nodes [2].
(2) *Network bandwidth consumption*: The size of visual data is much larger than scalar data so more network bandwidth resource are required for its transmission. As a result the bandwidth-demanding visual data transmission posts a challenging routing decision in limited frequency spectrum WVSNs. In addition, most of the applications require QoS (e.g., delay, jitter and loss) support so that the visual data could playback at the sink nodes successfully. These QoS requirements make the visual data transmission in WVSNs even more challenging than in WSNs.
(3) *Collision in transmitting data*: When an event occurs, neighboring sensors that detect the event will transmit the visual data back to the sink node at the same time. Because these neighboring sensors node are often geographically close to each other, collisions will happen when transmitting the data at the same time. In addition, due to the large visual data size, the sensors have to transmit a sequence of packets to be able to transmit the whole captured visual data. This will further increase the probability of collisions. When collisions or interference happens, the garbled packets need to be retransmitted. Such retransmissions will increase the possibility of collisions even more. In order to tackle this issue, new MAC aware transmission and routing schemes should be devised for WVSN.
(4) *Multimedia data processing*: As compared to the scalar data processing in WSNs, the visual data processing in WVSNs is more complex and it demands more hardware resources such as CPU power and memory buffer. In order not to consume so many hardware resources at the relay sensor nodes, one possibility is to reduce the size of the visual data. Shrinking the size of the visual data could be done either by compression techniques via a single sensor node or by multimedia processing methods to eliminate the redundant parts between multiple sensor nodes. Visual data compression via a single sensor is easy, but it comes with the cost of inferior visual data quality. Multimedia processing methods require collaboration between multiple sensors to eliminate the redundant parts in their captured visual data. The collaboration among sensor nodes needs to exchange FoV information and then sophisticated multimedia processing techniques must be devised to determine the redundant parts from the exchanged FoV information. The sensor hardware capability should be considered when designing the visual data processing techniques.
(5) *Sensor coverage in WVSN*s: In the WVSN, because of the relative angle or obstacles between the object and the sensor node, the camera on the sensor node might not be able to capture the desired shot even if the object is within the FoV of the sensor node. As a result some important scenes (e.g., face of the intruder or license plate of the car for security surveillance applications) might not be captured due to the limited angle of the camera or the obstacle blocking the view. This kind of occlusion problem requires more sensor nodes to be deployed. Devising methods to optimize the sensor deployment in occlusion-aware WVSNs so as to minimize the deployment cost is an interesting research problem in WVSNs.

As discussed in the previous paragraph, existing WSN algorithms or schemes are not applicable to WVSNs. Visual data introduce a new dimension to enable a much wider range of applications, but it also brings new research challenges and opportunities. New algorithms and schemes should be proposed to address the sensor coverage and visual data capturing/processing/transmission issues.

In Section 2, we survey existing works on the development of sensor hardware and platforms for WVSN networks. In Section 3, we assess existing works on sensor coverage and sensor deployment in WVSNs. In Section 4, we study existing works on visual data capture in WVSNs. In Section 5, we examine existing works on visual data processing in WVSNs. In Section 6, we survey existing works on visual data transmission. In Section 7, a new research paradigm based on social networking with WVSNs is surveyed. In Section 8, we conclude what has been done and not done in WVSNs.

## 2. Node Hardware Components in WVSNs

The hardware components of the visual sensor node consist of the image capturing device, processing unit, memory, radio and power supply. The image capturing device could be a CCD webcam (e.g., Meerkats [3], Panopes [4]) or a CMOS imaging device (e.g., Vision Motes [5], Cyclops [6]). The processing unit could be a non-programmable logic or a programmable logic unit. The radio module could be IEEE 802.15.4 (e.g., MeshEye [7], Cyclops [6]), Bluetooth (e.g., MicrelEye [8]) or WiFi (e.g., Meerkats [3]).

Basically, CMOS imaging devices are smaller and cheaper than CCD webcams, however, the image quality of CMOS is not as good as that of a CCD webcam. For example, the Cyclops [6] embeds the Agilent ADCM-1700 CMOS imaging device with a resolution 355 × 288. The Meerkats [3] equips the Logitech 4000 USB webcam with a resolution 640 × 480. In general, better image resolution means larger image size and implies more processing and transmission power. The tradeoff between the visual data quality and system resource consumption depends on the requirements of the application. For applications that require higher image quality (e.g., face recognition in security surveillance), a CCD webcam is a better choice. For applications that requires capturing many images but do not require high resolution (e.g., object tracking in wildlife observation), a CMOS is a better imaging device. Besides the requirements of the application, the imaging device selection depends on the tradeoff between the cost, size, power consumption and visual data resolution.

The non-programmable logic unit is usually designed for a specific application so it has better performance and lower power consumption. With mass production, the unit cost could be even lower. For an application that requires high volume of nodes, a non-programmable logic unit is preferable because of its low manufacturing cost. However, the drawback is that the processing unit cannot be altered to meet another function. In programmable logic units (usually FPGAs), the design can be altered for other application. In WVSNs, this flexibility is important because often one needs to change the parameters or the vision computation algorithm to obtained the optimized performance in different environmental settings (e.g., luminance). This flexibility makes programmable logic unit dominate the processing units in WVSNs.

The memory and storage requirements for a WVSN is much higher than for a WSN because the sensor nodes needs to capture, process and transmit visual data instead of scalar data. This stringent memory and storage requirement makes most of the existing WSN platforms not applicable for WVSNs. The well-known WSN mote, Mica2 [9] is equipped with 4 KB RAM and 128 KB FLASH to sense and process the scalar data. Cyclops [6] captures low resolution CIF (355 × 288) images to perform object detection tasks. It is equipped with 64 KB SRAM and 512 KB FLASH. For the standard VGA (640 × 480) image and 24 color bits for each pixel captured by the webcam, the image size is 921.6 K bytes without compression. The required memory and storage requirement is at least 2 MB to have enough space for image processing. Meerkats [3], has 32 MB FLASH and 64 MB DRAM that could process the VGA (640 × 480) images to perform object tracking. In summary, the memory requirements for the sensor nodes in WVSNs are at least ten times more than in WSNs.

In WVSNs, like in WSNs, most of the sensor nodes are powered batteries and it is often difficult to recharge or replace the battery on the sensor node. Therefore, energy efficient mechanisms have become important research topics in WVSNs to prolong the lifetime of the WVSN. Sophisticated energy efficient mechanisms rely on the understanding the battery discharging behavior in the WVSN. In [10–12], the detailed battery discharging process is well studied. First of all, the battery capacity is a measure of the total charge that can be extracted from a battery, usually in current time units (e.g., mAh) [10]. Note that the energy consumption is equal to the electric charge times the voltage. The voltage for most of the WVSN platforms is between 1.5 V to 3 V.

In [10], the power consumption on MICA2DOT platform is studied, and it is found that the radio power consumption dominates the other two modules (CPU processing and LED lighting). In addition, the battery lifetime for large transmission power (10 dBm) is 45% shorter than for small transmission power (0 dBM). With adaptive power control mechanism, the battery efficiency could increase 52%. In [11], it is also concluded that the adaptive power control could help to prolong the lifetime of the WSN.

In [13], the model of battery discharge characteristic in the WSN platform, TelosB, is used to predict the battery lifetime. During the pulse discharge of the battery, the operating voltage varied between an upper and a lower voltage [12]. In [13], the voltage variance of battery is captured in the model to predict the lifetime of the battery more accurately. As indicated above, all the works on the battery discharging behavior are for the sensor nodes in WSNs, and none of these works study power consumption for the camera sensors in WVSNs. To be more specific, the battery discharge behavior for image/video capturing, processing and transmission has yet to be explored.

The radio modules of WVSNs could be classified as low rate wireless personal area networks (WPANs) and high rate wireless local area networks (WLANs). IEEE 802.15.4 and Bluetooth are the typical WPAN radio modules in WVSNs. The transmission range of the WPAN radio modules is restricted to 10 to 20 m. Due to their small transmission range, they have lower power consumption. The maximum data rate for the IEEE 802.15.4 and Bluetooth is 250 Kbps and 1Mbps, respectively. IEEE 802.11b is another popular WLAN radio module in WVSNs. The maximum data rate of the 802.11b could be 11 Mbps and transmission range could reach 100 m. Because of its higher data rate, IEEE 802.11b radio modules in WVSNs are relevant to applications that require real-time video streaming (e.g., vehicle tracking). This higher data rate and longer transmission range comes at a price of higher cost and power consumption. For example, MeshEye [7] adopts the CC2420 radio chip, which is an IEEE 802.15.4-compliant RF transceiver. It consumes 175.9 mW in transmission and 1.8 mW in sleeping mode. On the other hand, Meerkats [3] adopts the IEEE 802.11b module. It consumes 3.5 W in transmission and 49.2 mW in sleeping mode, which is more than one order of magnitude higher than the power consumption in MeshEye [7].

In summary, until now, according to the computational capability and system resource consumption, there are two types of WVSN platform. The first one is the low end WVSN platform that is designed specifically for energy efficiency (e.g., Cyclops [6]). The other is the high end WVSN platform (e.g., Meerkats [3]) that is designed for sophisticated visual data applications where the system resource requirement and energy consumption are about one order of magnitude higher. This offers new research directions and opportunities for designing new WVSN platforms that could perform sophisticated visual data operations in energy efficient ways. In this section, we review the five major hardware components of the sensor node in WVSN, other detailed hardware architecture can be seen in [14]. In Table 1, we summarize the node components for the WVSN platforms.

## 3. Sensor Coverage/Deployment in WVSNs

**Figure 2.**
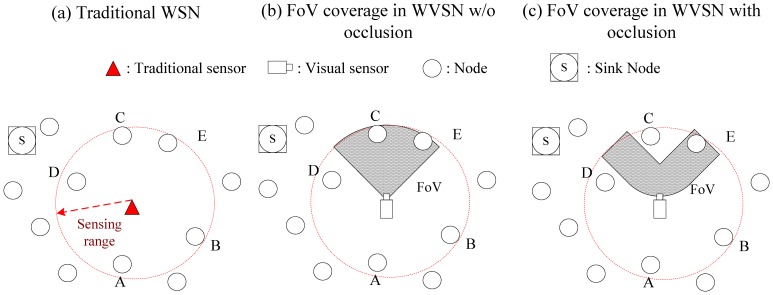
Sensor coverage in WSN and WVSN.

In WSNs, the sensors can cover an event if it is within sensing range. However, in WVSNs, besides the sensing range coverage, the event must also fall within the view angle of the camera on the sensor. Because the view angle of the camera equipped on the sensor node is limited, directional coverage instead of omni-directional coverage is applied in WVSNs. As indicated in Figure 2b, only two nodes (i.e., nodes *C* and *E*) are inside the coverage area of the camera sensor. Hence, there are two requirements (sensing range coverage and view angle coverage) for sensor coverage in WVSNs. The directional coverage literature in WSNs is surveyed in [15]. However, besides these two requirements, there is another factor (i.e., the occlusion problem) that needs to be addressed.

In the WVSN, the camera on the sensor node could only capture the images of the object without any obstacles. Hence, line-of-sight is required between the camera on the sensor and the object. If there is an obstacle (e.g., a tree) between the sensor and the object, the sensor cannot capture the object image even if the object is within the view angle coverage of the sensor. This is known as occlusion. To be more precise, the FoV coverage in WVSNs should consider the view angle of the camera and the occlusion at the same time. Hence, as compared to the sensor coverage problem in WSNs that only needs to consider the sensing range coverage, in the sensor coverage problem in WVSNs, there are three criteria to be considered (i.e., sensing range coverage, view coverage, occlusion).

In Figure 2, we show the differences between the sensor coverage problems in WSNs and WVSNs. In Figure 2a, the sensor could sense and capture the data from the nodes that are within sensing range. Hence, the sensor could capture the data from five nodes (i.e., nodes *A, B, C, D* and *E*). In Figure 2b, besides the sensing range, the view angle from the camera on the sensor nodes pose another constraint on the coverage. The FoV of the sensor covers nodes *C* and *E.* In Figure 2c, we could observe that the node *C* is behind the obstacle so that the FoV of the sensor could only cover node *E.*

Research on sensor coverage problems in WVSNs can be traced back to the art gallery problem. The art gallery problem is to identify how many guards are needed to guard an art gallery and how should they be placed [16]. There are polynomial algorithms to solve the 2-D art gallery problem. When the view angle of the camera on the sensor is 360° and the sensing range is unlimited, then the sensor coverage problem in WVSN could be reduced to the art gallery problem. However, in most of the cases, the view angle of the camera is less than 360° (e.g., 120°) and sensing range of the sensor is limited. This makes the sensor coverage problem in WVSN more challenging than the art gallery problem.

In [2], we consider a WVSN deployment algorithm with consideration of the sensing range coverage and angle coverage constraints. Optimization-based heuristics are proposed to tackle this problem. From the computational experiments, fewer sensors will be needed in a smaller grid size when in fixed sensing range and span angle. In [17], the angle coverage problem in WVSNs is assessed. When sensors are deployed, the objective is to identify a minimum set of sensors that can capture all the angles of view of the object while fulfilling the image resolution requirements. This paper is about an object tracking system capturing the images of moving targets from all angles.

In [18], a visual sensor deployment algorithm, which minimizes the total deployment cost while guaranteeing full multi-perspective (or multi-angle) coverage of the area (i.e., the coverage needed for video panorama generation) and the minimum required resolution is discussed. This multi-angle coverage problem is the same as the all angle coverage problem described in [17]. In both of these works, the occlusion problem is not considered.

References [19–21] discuss the sensor deployment problem with consideration of occlusion. In [19], by assuming the shape of obstacle to be square block, they derive the expected coverage area of the camera sensors. The mathematical derivation of the coverage area starts from one camera sensor and then generalizes to the multiple sensors. The assumption of square shape obstacle helps to derive the coverage mathematical model but it is not applicable to real WVSNs. This mathematical coverage model is a lower bound on the coverage areas because it fails to consider the possible cooperation between camera sensors to reduce the uncovered areas caused by the obstacle.

The method to calculate the “*certainty map*” that are the non-occluded areas for target localization and counting applications is found in [20,22]. By cooperatively fusing the certainty map from neighboring sensors, the occluded areas beside the objects and obstacles could be minimized. In [21], based on the idea of certainty map, the authors derive a close form visual coverage mathematical formulation that considers visual occlusions. Then, the minimum sensor density that suffices to ensure a visual K-coverage in a crowded sensing field is estimated.

In [23], the two-tier deployment problem in WVSN is considered. Tier-1 consists of visual sensor nodes that can capture the image data and tier-2 consists of relay nodes that can relay the image data back to the sink node. The goal is to minimize the deployment cost and at the same time to prolong the lifetime of the WVSN. However, without considering the FoV angle coverage and visual occlusion makes this work is not applicable to real WVSN networks.

In [24], a decentralized control strategy is proposed to position and orient the cameras placed on flying robots so as to cover the targeted area with minimum deployment cost. The control strategy considers heterogeneous degrees of mobility, where some cameras can translate and some cameras can only rotate. They propose an interesting performance metric, “minimum information per pixel”, to minimize the aggregate information per camera pixel over the environment. With this minimum information per pixel performance metric, the cameras' overlapped FoV could be minimized. This performance metric also minimizes the number of camera sensors that needs to be deployed.

In [25], they consider the mobile camera sensor coverage optimization problem as a repeated multi-player game. They propose distributed camera sensor coverage learning algorithms based on game theory to maximize the coverage and in the same time minimize the processing/energy cost. In this constrained exact potential game, each sensor will move and set its camera to optimize its coverage utilizing only the information from its utility values and last play actions. The two proposed algorithms are proven to be convergent in probability to a set of Nash equilibria and global optima of a certain set of coverage performance metrics (tradeoff between coverage and cost).

In Table 2, we summarize the existing works on sensor coverage and the WVSN deployment problem. This table also shows existing works that do not address the four research issues namely, view angle coverage, occlusion, visual data quality aware and energy aware at the same time. Therefore, it is a challenge to consider solutions to these four issues that will meet the application needs in a cost efficient way.

## 4. Visual Data Capture in WVSNs

Because of the limited view angle of the camera on the sensor node, it often needs several cameras in different orientations to fully cover the object. Given that the sensor nodes are randomly deployed in the field, to identify a minimum set of sensors needed to fully cover the object becomes a challenging issue. One trivial solution would be letting the sensor nodes whose FoV cover the object to transmit the visual data to the sink node. With this approach, there would be a lot of redundant visual information transmitted back to the sink. This comes with significant extra energy consumption and transmission bandwidth. Another possible solution is to identify the minimum number of sensors that can cooperatively cover the object from different orientations.

Camera correlation models are an active research topic in the WVSN field. Camera sensor nodes are correlated if their FoVs are overlapped. Depending on the relative angle between the correlated camera sensors nodes, the captured images between these correlated sensors could be redundant or supportive. Intuitively, when the relative angle is 0°, the correlated sensors catch the image with the same angle, and this implies that the images are redundant and only one copy of the image should be transmitted back to the sink node. When the relative angle is 180°, the correlated sensors catch the image from opposite directions, implying that the images are highly supportive (especially for security surveillance) and all the images should be transmitted back to the sink node. However, in most of the cases, the relative angle is not exactly 0° or 180°, so sophisticated methods should be devised to determine if the captured images are redundant or supportive.

In [26], a spatial correlation function is proposed to describe the degree of correlation for the images observed by cameras with overlapped FoVs. The correlation entropy function is defined to measure the amount of information provided by these cameras. Based on the correlation-based entropy function, the correlation-based camera selection algorithm is proposed to select the cameras to transmit the captured images back to the sink node. Hence, instead of choosing all the cameras whose FoV covers the object to transmit the visual data, the correlation-based selection algorithm in [26] could select fewer cameras with a sufficient amount of visual information. In [27], they compute the intersection area from the FoVs of multiple camera sensor nodes with a 3D directional sensing model. With this model, the computation of intersection areas could be reduced to calculating the relative position between the camera sensor nodes and it could be done distributedly.

The camera sensor node activation problem in two-tier WVSNs is considered in [28]. The first tier is the scalar sensor node that is used to detect the object and sent a signal to activate the camera sensor node in the second tier. Because of the restrictions on the camera lens, an object that is too close or too far from the camera will not be in focus. By setting the near line and far line in the FoV, only the camera sensor nodes whose good FoV (between the near line and far line in FoV) that can cover the object in focus will be activated. A similar idea of good FoV is also shown in [29]. It is the idea of generating look-up tables to identify the camera nodes with overlapping FoVs. However, the construction of look-up table is based on the distance between the object and the center of the FoV, and this information is difficult to get since the location of the object and the center of the FoV are difficult to determine accurately.

In [30], an intelligent camera sensor actuation mechanism is proposed to actuate (turn on) the least number of camera sensor nodes to reduce the redundancy in the multimedia data while still providing the necessary event coverage. Two-tier sensor node (scalar sensor and camera sensor) architecture is considered, where the camera sensors can utilize the information from the scalar sensor to determine the priority of actuation. Basically, a camera sensor node hearing a higher number of messages from the scalar sensors indicates that the event has a higher chance to be in the FoV of this camera sensor node and it should be given higher priority to be actuated. By exchanging the number of the scalar sensors and the FoV, neighboring camera sensor nodes could collaborate to determine who should be actuated to capture the image and transmit. However, occlusion is not considered in [30].

In WVSNs, when the deployed sensors are running out of batteries or the wind changes the camera orientation, the network topology will change. Then an interesting question is how to identify the collaborative camera sensors to capture the images of the interests efficiently in a changing WVSN topology. In image/vision processing technology, “camera calibration” could determine each camera's position, orientation, and focal length automatically. With these camera parameters, each camera sensor could locate its collaborative sensors to perform high level vision processing tasks (e.g., multiple object tracking) more efficiently. Radke *et al.* [31–34] proposed distributed camera calibration algorithms to estimate the camera's position, orientation, and focal length. Then a vision graph is constructed to identify the overlapped FoV relations for the camera sensors. An edge on the vision graph represents two cameras that image a sufficiently large part of the same environment. By using the vision graph, when an event occurs, a set of collaborative camera sensors of FoV covering the event could be identified efficiently to perform high level image/vision processing.

A similar vision graph idea is also shown in [35], where a mathematical model is proposed to analyze the overlapped FoVs among the camera sensor nodes. For any sensor node, the probability of having a vision graph neighbor (i.e., the neighboring with the overlapped FoV) with respect to the distance between these two sensors is determined. With this vision graph neighbor information, it could identify the set of sensors that should capture the visual data cooperatively.

In WVSNs, the deployed camera sensors might not be able to cover the images of interest because of occlusion. In [36], virtual view image generation in WVSNs is studied. The virtual view image is generated when a user wants to see a particular object which is not within the FoV of any deployed camera sensor. Two visual sensors with opposite location and with smallest disparity criteria from the virtual view are selected. However, the generated virtual image quality is not examined carefully to justify this camera selection method.

In contrast to the image capture in WVSNs, the video capture in WVSNs consumes more resources in terms of processing power, memory and power consumption. In addition, the stringent delay constraints incurred from real-time video applications (e.g., security surveillance) makes video capturs and processing more challenging than that of images. In recent works, to meet the real-time constraint, centralized algorithms are developed to control the camera sensors to capture the video stream. In [37], a real-time video surveillance application where the captured video is sent back to the centralized Remote Control Unit (RCU) to send the pan or tilt command to control the cameras for target tracking is described. In order to meet the real-time requirement, instead of identifying a minimum number of cameras for FoV coverage, the goal is to identify the delay-constrained camera control mechanism on the RCU to control cameras for target tracking. In [38], an application-independent task mapping and scheduling mechanism in multi-hop WVSNs is studied to meet the real-time guarantees. The proposed algorithm is based on a Directed Acyclic Graph (DAG) that jointly schedules communication and computation tasks of an application with minimum energy consumption subject to delay constraints. However, in this work, the basic assumption of the task scheduling algorithm is that these tasks are independent. Such an independence assumption is valid in scalar data in WSNs, but not in visual data in WVSNs. In WVSNs, the energy consumption and visual data processing time for the tasks in the WVSN depend on the correlation among the camera sensor nodes.

In Table 3, we summarize the existing works on visual data capture in WVSNs. In most of the existing research works, the camera sensor node is semi-static (i.e., cameras can only pan and tilt). However, in the near future, new Mobile, Pan, Tilt and Zoom (MPTZ) camera sensors that can move and adjust the camera on-the-fly will be available. This opens a new visual data capturing era in WVSN networks. This MPTZ camera sensor enables fewer sensors to be randomly deployed at the initial stage and then the camera sensor can be moved and adjusted to meet the application needs. This brings up new research opportunities on visual data capturing by using MPTZ camera sensors to meet the application requirements in an energy efficient way.

## 5. Visual Data Processing in WVSNs

In WVSNs, the visual data includes image and video. The richer content of the visual data makes the visual data processing more challenging than the scalar data processing. Multimedia processing has been an active research field for decades. Feature extraction and processing are the most popular methods used in multimedia processing. The features extracted from the image data include the color, texture and shape. The extracted features from the video data include the motion features that are computed from the pixel variation from consecutive video frames. In multimedia processing, multimedia fusion is also an active research field [39]. It is discussed in [39] that the visual data from multiple camera sensors should be fused and processed in WVSNs. The idea of these traditional multimedia fusion methods is on fusing the different formats of multimedia data to perform certain tasks (e.g., human tracking). These traditional multimedia fusion methods do provide some basic ideas for visual data processing in WVSNs, but traditional multimedia fusion methods are not applicable in limited battery power and limited bandwidth WVSNs.

Besides the visual data fusing technique, another interesting technique in visual data processing is image/vision aggregation. In WVSNs, when an event occurs, neighboring camera sensors might capture images/video with high similarities. If the images/video could be combined to eliminate the redundant portions, then system resource (battery and bandwidth) consumption could be reduced. This is known as image/video aggregation. Video aggregation could be realized by multi-view video coding techniques. In [40], the efficiency of multi-view video coding (MVC) and single-view video coding (SVC) in WVSNs are compared. By leveraging the spatial correlation among partially overlapped FoVs, the MVC has advantages in terms of compression efficiency so that the total bandwidth consumption could be reduced. The authos conclude that when there is more than 50% overlapped area and the angular displacement is less than 15 degrees, MVC outperforms SVC.

In [41], the idea of image aggregation is realized by using the Square JPEG (S-JPEG) mechanism to reduce the redundant data in the sent images by using the concept of “Reduced Block Size” in the two-dimensional discrete cosine transform. The S-JPEG is shown to be more energy efficient than the standard JPEG. However, the image quality could be compromised at the sink, hence, there is a tradeoff between image quality and energy consumption. As compared to JPEG, S-JPEG is more suitable for applications with stringent energy constraints but which do not need a full image quality at the sink (e.g., security surveillance).

In [42], the image compression scheme in WVSNs is also studied. An information theoretic image compression framework with the objective of maximizing the compression of the visual data captured in WVSNs is proposed. In this framework, camera sensor nodes are grouped into clusters where each cluster has a cluster head that performs the data encoding. With this kind of clustering, the sensor node could alleviate the problem of computational intensive operations and the cluster head is responsible for handling the correlation between the received images from the sensors in the cluster. The novelty of this image compression framework is that it is independent of the image types and coding algorithms. From the simulations, they show that the proposed compression framework could save 10% to 23% total coding rate as compared to the scheme where each camera sensor compresses its own image independently.

Even though image/video aggregation looks like a promising way to deal with the battery-limited and bandwidth-limited transmission in WVSNs, image/video aggregation requires image/video processing that incurs node processing power. In addition, there is extra MAC layer retransmission energy loss from image/video aggregation. In [43], they study the interplay of these three factors (image transmission, image processing and MAC retransmission) is studied for image aggregation in WVSNs. It is concluded that image aggregation should be encouraged to decrease the total power consumption in highly redundant portions of the captured images and in a large transmission radius. When there are more than seven aggregated data source nodes, the MAC retransmission losses will dominate the other two factors so that image aggregation should not be encouraged.

Novel visual data processing schemes require good quality visual data in WVSNs. Methods to determine and measure the quality of the captured visual data are important and challenging. The quality of sensor-driven multimedia information in terms of certainty, accuracy/confidence and timeliness are defined in [44]. A model is proposed to characterize these performance metrics. Then, these three performance metrics are computed dynamically to determine the quality of information based on current sensor observation, agreement/disagreement among the participating sensors, current context information and prior confidence of the sensors in performing various detection tasks. This mechanism could be used in a real-time application (e.g., security surveillance) where the ground truth is not available to verify the observation.

To prolong the lifetime of WVSNs, besides energy efficient data processing schemes, energy efficient visual data transmission schemes should also be devised. In [45], they jointly consider energy efficient data processing and visual data transmission schemes. A mathematical model to capture the interplay between source rates, encoding power, routing, video quality and network lifetime is developed. The distributed algorithm is proposed to maximize the network lifetime by jointly optimizing the source rates, the encoding powers, and the routing scheme. Transmission errors are considered in [45] and error remedy techniques in both large delay applications (e.g., environmental data collection) and small delay applications (e.g., real-time traffic monitoring) are studied to quantified the impacts on the maximum network lifetime.

Because of the limited hardware capability of the camera sensors in WVSNs, collaborative and cooperative visual data processing among the camera sensors is necessary to meet the application requirements for visual data quality, delay time, packet loss and power consumption. As recalled in Section 4, the captured images between the neighboring sensors could be redundant or supportive. Intelligent cooperative visual data processing schemes should ignore the redundant images and only process the supportive images. However, this is not an easy task since it involves not only the relative angle and locations of the camera sensors, but also needs to meet the requirements of the application. Existing researches shed some light on the collaborative and cooperative visual data processing techniques, but there is still a big room for improvements.

In Table 4, we summarize the existing works on visual data processing in WVSNs. Most of the existing works borrow the idea from traditional multiprocessing techniques. Even though image/video processing has been studied for decades this traditional multimedia processing could not be directly applied in WVSNs because of the limited resource constraints on processing capability, battery power and wireless bandwidth. In meeting the challenges of limited resources, new novel collaborative visual data processing schemes among the sensor nodes should be developed.

## 6. Visual Data Transmission in WVSNs

Visual data transmission in WVSNs is very expensive due to its requirements of large amounts of bandwidth and battery power. Hence, as compared to the routing issues in WSNs, we have more stringent battery and bandwidth constraints in WVSNs. In [46] a routing scheme for the image sensors to route the captured image data to the base station is proposed. The routing decision is based on the three parameters (relative position to the base station, queue length and residual power) of next hop neighboring relay sensors. They study the tradeoff between these three parameters to optimize the system throughput and prolonging the network lifetime.

Reference [47] proposes delay constrained routing in WVSNs. The basic idea is to route the highly relevant visual data through faster paths with lower end-to-end delay. The discussion on reactive opportunistic routing scheme to balance between power consumption and delay in [48] is a similar idea. The basic idea is to choose the next relay node based on the location information and packet relevance information. High relevance packets choese the shorter path to the sink node and low relevance packets use the leftover paths that have enough battery power.

These three works assume that the relevance of the captured visual data is fixed or given. Even though this assumption could simplify the problem complexity, it is not applicable to real-time WVSN applications since the relevance of visual data changes from time to time based on the camera's FoV and the application needs. In other words, the routing decisions should also consider the camera's FoV and the application needs.

Some of the works consider the data capture and transmission simultaneously. In [49], they propose a distributed visual data capture and routing algorithm for WVSNs subject to energy constraints. The basic idea of the algorithm is to allocate the energy in data capture (sampling) and routing based on exchanging the collected visual data and residual power information with neighboring sensor nodes. They compare two routing schemes (fixed routing and flexible routing) under the same power budget constraint. They found that as compared to the fixed routing scheme, flexible routing could deliver twice the amount of visual data back to the base station but it comes at about 100 times the communication and computation cost. They conclude that a flexible routing energy allocation scheme is applicable only in small networks. In [50], they study the inter-node differential coding and routing issues simultaneously to reduce the captured overlapped FoV data transmitted on the WVSN. Their basic idea is to cluster the sensor nodes whose FoVs are overlapped and then the routing decision is based on cluster information and distance to the sink nodes. Since the FoV of the sensor nodes in the same cluster is overlapped, then relaying the visual data via the same cluster member with shorter distance to the sink nodes could reduce the visual information injected into the network.

Ant-based WVSN routing protocols are proposed in [51,52]. The basic idea of the ant-based routing protocols is to mimic the behavior of the ants searching for the food through pheromone deposition in the sense that future ants could locate food using the pheromones left behind by previous ants. By adopting this idea, ant-based routing protocols use reinforcement learning algorithms to locate the QoS aware [51] and energy efficient path [52] to the sink nodes. The reinforcement algorithm is to encourage the route that is close to the sink and discourage the routes that stray from the sink. Ant-based routing is a promising routing protocol in *ad hoc* networks that it incorporates the good features of fully distributive, simple operations in the node, fault tolerant, adapt to changes in network topology and traffic demands [53]. We believe that ant-based routing protocols will also play an important role in resource limited WVSNs. However, ant-based routing protocols in WVSNs are still in the early stages and more research efforts are needed to tackle the problem of large bandwidth applications in limited resource WVSNs.

**Figure 3.**
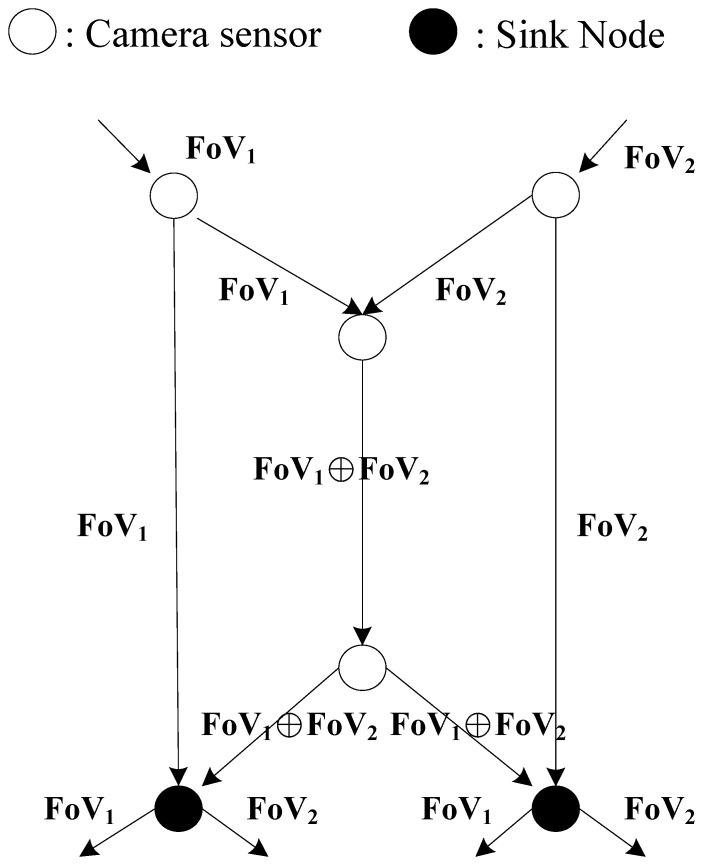
Network coding in WVSN.

Network coding is another promising technique to save the bandwidth transmission for routing in networks so that network coding could be a powerful routing scheme in bandwidth limited and energy limited WVSNs. The basic idea of network coding is to allow the mixing of the data at the intermediate nodes and then the original data could be extracted from these mixed data at the receivers. In [54], they propose a famous butterfly example to demonstrate that network coding could outperform routing. In Figure 3, we indicate this butterfly example in a WVSN. Two captured FoV datasets (i.e., FoV_1_ and FoV_2_) from different camera sensor nodes are transmitted on the network to the sink nodes. If the capacity on each link can carry one FoV dataset, then traditional routing schemes would fail to deliver these two FoV datasets to the sink nodes because of the capacity limitations in the central link. Even if there is no capacity constraint, the central link still needs to transmit two visual datasets (i.e., FoV_1_ and FoV_2_) so that the sink nodes could reconstruct the visual field. However, by using the network coding scheme, by transmitting the combined FoV data (i.e., FoV_1_⨁FoV_2_) at the central link, the sink node is able to deduce the original two FoV datasets via simple XOR operations. For example, FoV_2_ = FoV_1_⨁FoV_1_⨁FoV_2_, then the left sink node can deduce the FoV_2_ data from a simple XOR operation. Similar XOR operations could be performed at the right sink node to deduce the FoV_1_ data. Since the XOR operation is a simple bitwise operation, the power consumption on XOR operation is much smaller than transmitting two visual data on the central link. Then the sink nodes could reconstruct the visual field with more bandwidth and in an energy efficient way. Future research works are encouraged to adopt the idea of network coding to transmit the visual data in WVSNs.

In [55,56], the idea of network coding is adopted in their proposed routing schemes. In [56], a joint coding and routing scheme for WVSNs is proposed to optimize the tradeoff between network lifetime and video quality (distortion). First of all, they adopt the WZ-based multi-view video coding scheme [57] to encode the captured multi-view video streams into K or WZ frames. Then video quality could be measured by the video distortion of the received K and WZ frames. Second, network coding is adopted to split the captured video stream into multiple sub-streams via multiple routes to the sink nodes. A general energy model is proposed to capture these two types (i.e., video coding and multi-path routing) to maximize the network lifetime and in the same time to approximately reconstruct the visual field at the sink nodes.

In Table 5, we summarize the existing works on visual data transmission in WVSNs. Here we observe that none of the existing works address the delay, visual data quality and power consumption at the same time. In general, smaller transmission power could save battery power, but it also sacrifices the visual data quality at the receiver. End-to-end delay composes of queuing delay, node processing delay and retransmission delay at the MAC layer. Node processing delay in WVSNs comes mostly from the combined FoV calculation. Hidden node problem in the wireless network introduces additional retransmission power loss and delays due to the collisions at the aggregated nodes. This makes the delay-constrained routing algorithms in WVSNs more challenging than in WSNs or other wireless networks. Tradeoff between these three criteria in making the routing decision poses interesting questions for researchers.

## 7. Social Networking Paradigms in WVSNs

From the previous sections on sensor deployment, visual data capture and processing, it is evident that due to problems like sensing range limitation, angle limitation, and occlusion a single camera sensor node might not capture the visual data of the target. Therefore the visual data reconstruction depends on the cooperation among the camera sensor nodes to meet the system resource consumption and visual data quality requirements of the applications. Good cooperation strategies between the camera sensor nodes need to consider the requirements of the applications and the correlation between the visual data captured by the camera sensor nodes. As existing research works on sensor node cooperation and visual data correlated fusion are preliminary, more novel research results are needed in this area. Besides the conventional research methodologies on image/video processing and WSNs, new research methodologies and paradigms should be proposed to tackle interesting issues in WVSNs. One of the new promising research paradigms is to leverage on the idea of social networking.

An interesting research methodology is presented in [58], called Social Internet of Things (SIoT), on the Internet of Things (IoT), to manage the interaction of objects in terms of the social behavior and relationships by using the social networking research works by Fiske [59]. In [59], Fiske proposed four social relational models (communal sharing, equality sharing, authority ranking and market pricing) to describe the social interactions among human beings. The basic idea of “communal sharing” is that each entity is individually indistinctive to contribute on generating overall interests. This kind of relationship is also shown in WVSNs where each camera nodes are indistinctive from each other to capture and process visual data to meet requirements of the applications. The basic idea of “equality sharing” is that there are egalitarian relationships among the entities characterized by in-kind reciprocity and balanced exchange. This kind of relationship is also shown in WVSNs where the visual data is exchanged equally between the camera nodes. The basic idea of “authority ranking” is that relationships are asymmetrical among the entities based on the precedence, hierarchy, and status. This kind of relationship is also shown in WVSNs when there are node hierarchies where camera sensor nodes transmit the captured unprocessed visual data to the camera sensor nodes with more hardware capability to do further visual data processing. The basic idea of “market pricing” is that there is relationship based on proportionality, with interactions organized with reference to a common scale of ratio values. This kind of relationship is also shown in WVSNs where the visual data is exchanged between the camera sensor nodes.

In [58] and [60], based on the social networking concept in [59], a new SIoT architecture with a IoT server and client is devised. Hence, the processes of the four main SIoT activities (new object entrance; service discovery and composition; new object relationship; service provisioning) could be defined and implemented. Based on the architecture and the activities, the capability of human and devices to discover, select and use objects with their services is also augmented in the IoT. This leaves one interesting open question to be answered: could SIoT architecture be directly applied in WVSNs? If not, what is the correct social networking architecture for WVSNs? More research is needed to answer this interesting open question.

According to the previous research results, social networking is a promising approach in large scale IoT. We believe that social networking would also be another novel research paradigm in tackling the interaction between the camera sensor nodes. By leveraging the interaction, intelligent collaborative and cooperative sensor coverage, capturing and processing schemes could be developed. Essentially, ways of adopting social networking research idea in WVSNs so that the camera sensor nodes could discover and build relationships with other camera sensor nodes to deliver the QoS-aware visual data in a more economical way should be devised.

## 8. Conclusions

WVSNs are next generation WSNs that enable more visual data-based applications, such as security surveillance, home care and environment monitoring. On the other hand, WVSNs also introduce new challenges and research opportunities. First, instead of sensing range coverage, the FoV coverage in WVSNs is determined by the camera's view angle, focal depth and occlusion caused by the obstacles. Second, visual data is much bigger and more complicated than scalar data. Processing and transmitting visual data consumes much more resources than scalar data. Third, visual data reconstruction at the sink node relies on the cooperation among multiple correlated data sources nodes to reconstruct the whole picture of the interested objects. Fourth, the QoS requirements (e.g., resolution, delay, jitter, packet loss) for the visual data applications are more stringent than for scalar data applications. These four new challenges make the traditional WSN algorithms or protocols inapplicable to WVSNs. Existing researches in WVSNs are still at an early stage and there are still many open issues that need to be addressed in the aspects of sensor coverage, visual data capturing/processing/transmission. We believe that novel WVSN algorithms or protocols should be a multi-disciplinary (i.e., multimedia processing, wireless communication and networking, distributed processing and intelligent system) and cross-network-layered design.

## Figures and Tables

**Table 1.** Hardware components in WVSN platforms.

**Platform**	**Image Capturing Device**	**Memory and Storage**	**Radio**	**Energy Consumption (mW)**
Cyclops [6]	CMOS imaging device	64 KB SRAM, 512 KB FLASH	Zigbee	0.8–110.1
Meerkats [3]	CCD webcam	32 MB FLASH and 64 MB DRAM	IEEE802.11b	49.2–3,500
Panopes [4]	CCD webcam	64 MB	IEEE802.11	58–5,300
MeshEye [7]	CMOS imaging device	64 KB SRAM, 256 KB FLASH	Zigbee	1.8–175.9
MicrelEye [8]	CMOS imaging device	32 KB SRAM, 1 MB FLASH	Bluetooth	∼500
Vision Motes [5]	CMOS imaging device	64 MB SRAM, 128 MB FLASH	Zigbee	5.2–489.6

**Table 2.** Research works in WVSN deployment.

**Research Works**	**Camera View Angle**	**Visual Occlusion**	**Vision Quality Aware**	**Energy Aware**	**Notes**
Chow *et al.* [17]	Yes	No	Yes	No	Full multi-angle coverage
E. Yildiz *et al.* [18]	Yes	No	Yes	No	Full multi-angle coverage
Y.-T. Lin *et al.* [19]	Yes	Yes	No	No	Square shape obstacle assumption
M. Karakaya *et al.* [20–22]	Yes	Yes	No	No	Cooperatively certainty map fusing
H. Li *et al.* [23]	No	No	No	Yes	Two-tier WVSN network deployment
H. H. Yen [2]	Yes	No	No	No	Sensing range and angle coverage
Schwager *et al.* [24]	Yes	No	No	No	3D mobile camera sensor control strategy
Zhu *et al.* [25]	Yes	No	No	Yes	Game theoretic sensor coverage model

**Table 3.** Research works on visual data capture in WVSNs.

**Research Works**	**Sensors Hierarchy**	**Collaboration**	**Gps Needed**	**Distributed Processing**	**Notes**
R. Dai *et al.* [26]	Single tier	Among camera sensors	No	Yes	Visual correlation entropy framework
C. Han *et al.* [27]	Multi-tier	Among camera sensors	Yes	Yes	3D directional sensing model
H.S. Aghdasi *et al.* [28]	Multi-tier	Scalar and camera sensors	No	No	Supervised learning needed
J. Park *et al.* [29]	Single tier	No	Yes	No	Distance-based lookup table
A. Newell *et al.* [30]	Two tier	Scalar and camera sensors	No	Yes	Counting the activated scalar sensors
R. Radke *et al.* [31–33]	Single tier	Among camera sensors	No	Yes	Estimate camera's position, orientation and focal length
X. Dong *et al.* [35]	Single tier	Among camera sensors	Yes	Yes	Vision graph construction
D. Wu *et al.* [37]	Single tier	No	No	No	Real-time camera control
Y. Gu *et al.* [38]	Single tier	No	No	No	Task mapping and scheduling mechanism

**Table 4.** Research works on data processing in WVSNs.

**Research Works**	**Processing Technique**	**Visual Quality Aware**	**Power Aware**	**Distributed Processing**	**Notes**
S. Colonnese *et al.* [40]	Video aggregation	No	No	Yes	Bandwidth efficient multi-view video coding
P. Wang *et al.* [42]	Compression framework	No	Yes	Yes	Correlation processing and coding via clustering
M. A. Hossain *et al.* [44]	Visual data model	Yes	No	Yes	Data quality determined by sensors collaboratively
A. Mammeri *et al.* [41]	Image aggregation	No	No	No	S-JPEG to reduce the redundant block size
Y. He *et al.* [45]	Optimizing source rates and encoding powers	No	Yes	Yes	Processing and routing schemes to prolong network lifetime
P. K. Atrey *et al.* [39]	Multimedia fusing	No	Yes	Yes	Fusing the different formats of multimedia
Y. L. Chen *et al.* [43]	Image aggregation	No	Yes	Yes	Energy efficient aggregation schemes

**Table 5.** Research works on data transmission in WVSN.

**Research Works**	**Delay Aware**	**Visual Quality Aware**	**Power Aware**	**Distributed Processing**	**Notes**
L. Savidge *et al.* [46]	No	No	Yes	Yes	Routing decision based on location, queue length and residual power
J. Kho *et al.* [49]	No	No	Yes	Yes	Energy allocation scheme in sampling and routing
Costa *et al.* [47]	Yes	No	No	No	Priority routing based on the relevance and delay
Spachos *et al.* [48]	Yes	No	Yes	Yes	Data relaying based on power and relevance
Li *et al.* [56]	No	Yes	Yes	Yes	Joint multi-view video coding and multipath routing scheme
Cobo *et al.* [51]	Yes	No	No	Yes	Ant-based routing protocols
Zungeru *et al.* [52]	Yes	No	Yes	Yes	Ant-based routing protocols
Dai *et al.* [50]	Yes	No	Yes	Yes	Joint video coding and routing protocols
